# Vestiges of Creation

**Published:** 1846-02

**Authors:** 


### VESTIGES OF CREATION.

A third edition of this fashionable work has been called for by the American reading public, and is issued by Wiley & Putnam in a beautiful dress. These gentlemen have shown a becoming care for the orthodoxy of their countrymen by sending abroad the antidote bound up with the poison, in the shape of an admirable critique, from the North British Review. The former edition contained a noble defence of the truth, in a preliminary article; this gives the *coup de grace* to the sophistry of the Vestiges in an appendix. We examined the doctrines of this specious volume at considerable length, some months ago; since writing our article we have met with the following anecdote respecting it in an Edinburgh paper, with which we commend it to the curious of our readers, as a "scientific romance" of rare interest:

"We have been told that on its first appearance it formed the subject of a rather curious conversation between three scientific gentlemen,—a zoologist, a geologist, and an astronomer. 'Wonderful book!' said the astronomer, 'it reads like a novel, but its astronomy is wretched.' 'Say you so!' exclaimed the geologist, 'why I read it with very great interest indeed, and found nothing very bad in it except its geology.' 'And I,' said the zoologist, 'have also read it with interest, and liked its geology and astronomy very much, but found its zoology mere nonsense.'"

